# Joint integrity evaluation of laser beam welded additive manufactured Ti6Al4V sheets

**DOI:** 10.1038/s41598-022-08122-2

**Published:** 2022-03-08

**Authors:** P. O. Omoniyi, R. M. Mahamood, N. Arthur, S. Pityana, S. Skhosane, Y. Okamoto, T. Shinonaga, M. R. Maina, T. C. Jen, E. T. Akinlabi

**Affiliations:** 1 Mechanical Engineering Science Department, University of Johannesburg, P. O. Box 524, Johannesburg, South Africa; 2 Mechanical Engineering Department, University of Ilorin, P. M. B. 1515, Ilorin, Nigeria; 3 Department of Materials and Metallurgical Engineering, University of Ilorin, P. M. B. 1515, Ilorin, Nigeria; 4 National Laser Centre, CSIR, P. O. Box 395, Pretoria, South Africa; 5 Graduate School of Natural Science and Technology, Okayama University, Okayama, Japan; 6 Department of Mechatronics Engineering, Jomo Kenyatta University of Agriculture and Technology, 62000-00200 Nairobi, Kenya; 7 Pan Africa University for Life and Earth Sciences Institute, 200132 Ibadan, Nigeria

**Keywords:** Engineering, Materials science

## Abstract

The feasibility of joining laser metal deposited Ti6Al4V sheets using laser beam welding was investigated in this article. The additive manufactured sheets were joined using a 3 kW CW YLS-2000-TR ytterbium laser system. The mechanical properties and microstructure of the welded additive manufactured parts (AM welds) were compared with those of the wrought sheets welded using the same laser process. The welds were characterized and compared in terms of bead geometry, microhardness, tensile strength, fractography, and microstructure. The differences in characteristics are majorly found in the width of the bead and tensile strength. The bead width of AM welds appear wider than the wrought welds, and the wrought welds exhibited higher tensile strength and ductility than the AM welds.

## Introduction

The commencement of the fourth industrial revolution (4IR) has seen the growth of additive manufacturing (AM) or 3D printing skyrocket. AM applies 3D design data set to form a layer-by-layer deposition of material^1^; it is of great advantage over traditional and non-traditional machining techniques involving material removal. AM process results in less material wastage, as high power sources melt the powder to form three dimensional (3D) objects^2^. The AM technique also facilitates easy repair of components flexible fabrication of intricate and complicated shapes. Despite the numerous advantages of AM processes, it also has some limitations, such as confined build chamber size, which might not accommodate large parts production, and poor surface finish of product^3,4^. These limitations, therefore, brought about producing parts in smaller sizes and afterward joining them using fusion welding.

Laser metal deposition (LMD) is an AM process and a near-net-shape technology used to produce solid components. The process involves charging metallic powder or wire, which is melted by laser to form a melt pool, which then solidifies to form a metal deposit^2^. According to Segerstark et al.^5^, LMD can be categorized based on the feedstock used during manufacturing; powder and wire are the feedstocks used generally in LMD. In powder LMD (LMD-P), the powder is blown coaxially along with the inert gas into the melt pool, which might be argon or helium or a combination of both gases in most cases. On the other hand, wire is fed into the melt pool to create LMD-W^6^. Indeed, LMD has been helpful in the manufacturing industry. The aerospace industry has achieved an excellent buy-to-fly ratio with the LMD process, and the repair of components has also been achieved^7,8^. Titanium and its alloys have also been beneficial in the fourth industrial revolutionized industries due to its lightweight to strength ratio and excellent corrosion resistivity^9^. The aerospace industry has used titanium and its alloys to design and manufacture fuselage, turbine blades, shafts, and wings. Also, due to its biocompatibility, it is used as surgical materials and implants^10^.

Works of literature are limited on the mechanical and microstructural behavior of Ti6Al4V AM welds. Tavlovich et al.^11^ studied the mechanical and microstructural properties of AM welds using laser beam welding (LBW) and electron beam welding (EBW). The Ti6Al4V additive manufactured products were manufactured using selective laser melting (SLM) process. The AM welds were compared with wrought welds of Ti6Al4V in terms of tensile strength, weld bead profile, and microhardness. The boundaries of the fusion zone (FZ) were reported to be the significant difference between the welding techniques. The FZ of AM welds were wider and appeared to be straight compared to wrought sheets that show an hourglass shape.

In their research, Sun et al.^12^ used laser welding to join electron beam melting (EBM) manufactured Ti6Al4V product to wrought Ti6Al4V, the effect of welding angle on the mechanical properties and microstructure were studied. The microhardness measured was generally influenced by the local microstructure. The tensile strength was greatly affected by the base material (BM) of the additive manufactured product due to the defects developed primarily during the EBM process. Welding angle was observed to have an insignificant effect on the tensile properties of the welds. Conversely, there was a reduction in elongation as the welding angle reduced from 0 to 45°. Similar to the observations of Sun et al.^12^, porosities and non-isotropic properties are significant characteristics of AM parts that contribute significantly to the mechanical strength of AM welds. Besides, increasing energy per unit weld length is required to obtain a keyhole weld for AM welds compared to wrought Ti6Al4V welds^13^. Works of literature have also shown that the tensile strength of the AM welds is greatly affected by the additive manufacturing process. Titanium and its alloys are highly sensitive to atmospheric gases such as nitrogen and oxygen at a temperature of 350 °C and above^14^. These contaminations caused by atmospheric gases have been reported to affect the microstructure of welds. Coarse columnar grains and α’ martensitic microstructure have been reported to be responsible for brittleness, high microhardness, and reduced ductility^15^. Therefore, the ductility and toughness of welds and additive manufactured products can be improved through heat treatment^11,16^.

Laser welding (LW) is preferred in areas where high strength is required, such as the marine and aerospace industries. The smaller area of the weld zone (WZ) as a result of low heat input and high welding speed, compared to other fusion welding techniques such as metal inert gas (MIG) welding and tungsten inert gas (TIG) welding has made it advantageous^15,17^. Even though Ti6Al4V additive manufactured parts have been welded using different fusion processes as reviewed, the parameter selection has not achieved full penetration in some cases. Therefore, this study aims to substantiate the impact of laser power and welding speed on AM welds' mechanical properties and microstructure. The additive manufactured products were welded using laser welding, and the results were compared to wrought sheets welded using the same welding technique and parameters. All welds were joined in the butt arrangement.

## Materials and methods

**Figure 1:**
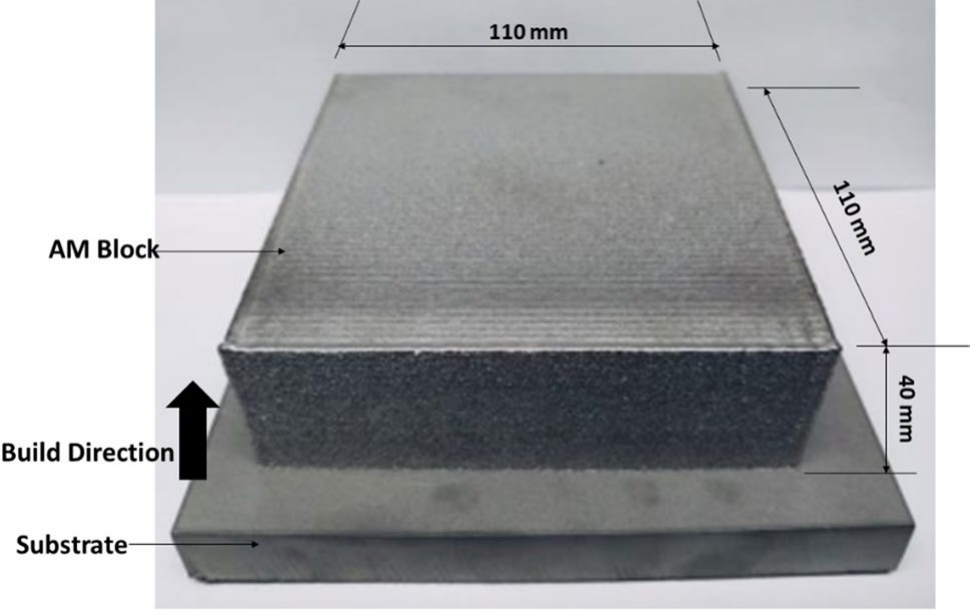
Additive manufactured product test specimen.

The wrought Ti6Al4V (grade 5) sheets used in this research were supplied by Saetra (PTY) Limited, Pretoria, South Africa, in mill annealed state. The dimension is 100 mm × 60 mm × 2 mm, and chemical composition in accordance with ASTM B265^18^ is shown in Table 1. The Ti6Al4V (grade 5) powder 45–90 µm and chemical composition in accordance with ASTM B215^19^ was supplied by WearTech (PTY) Limited, South Africa. The additive manufactured product (LMD) shown in Fig. 1 was built using Optomec 850-R LENS system, available at laser center of the council of scientific and industrial research (CSIR), Pretoria, with laser power of 400 W, powder feed rate of 2.4 g/min, hatch spacing of 0.9652 mm, and scanning rotated at 90° between successive layers. The AM block was then cut into thicknesses of 2 mm using wire electrical discharge machining (EDM).

**Table 1:** Elemental composition of wrought sheet and powder Ti6Al4V.

	Element	Ti	Al	V	C	Fe	N	O	H	Others
Wrought Ti6Al4V	Weight (%)	Remainder	6.10	4.0	0.03	0.15	0.018	0.13	0.002	Each < 0.10
Powder Ti6Al4V	Weight (%)	Remainder	6.44	4.06	0.01	<0.01	0.02	0.10	0.002	Each < 0.10

### Laser welding of parts

**Figure 2:**
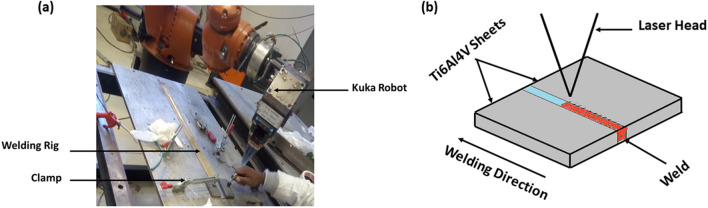
(**a**) Experimental setup, (**b**) schematics of the experimental setup.

The faying sides of the materials were cleaned with acetone to remove impurities before welding. 3 kW CW YLS-2000-TR ytterbium laser system available at laser center CSIR, Pretoria was employed in welding the parts autogenously. A special welding rig was also employed, with the ability to back purge to prevent contamination of the weld, as shown in Fig. 2a and the schematics of laser welding in Fig. 2b. The shielding gas used was argon with a flow rate of 15 L/min. The same welding parameters used for welding the wrought sheets and the AM products are presented in Table 2.

**Table 2:** Experimental process parameters.

S/N	Laser power (kW)	Welding speed (m/min)	Energy input (J/mm)
L21, AM21	2.8	2.8	60.00
L22, AM22	2.8	2.6	64.62
L23, AM23	2.7	2.8	57.86
L24, AM24	2.7	2.6	62.31

*L denotes wrought laser welds, AM denotes AM welds.

The energy input is calculated using equation (1)^3^.

1 $$E = \frac{P}{\nu }$$

where: Energy input *E* (J/mm), Laser Power *P* (Watt), Welding speed *v* (mm/s).

### Sample preparation and characterizations

Samples measuring 25 mm × 10 mm × 2 mm were cut out of the welded sheets across the welds. Each sample was mounted on thermoset resins then the grinding was done using SiC papers (#320-#1200). They were further polished till a mirror surface was achieved ASTM E3^20^. Kroll’s reagent was used in etching the surface of the weld for 18 seconds, in accordance with ASTM E407^21^.

**Figure 3:**
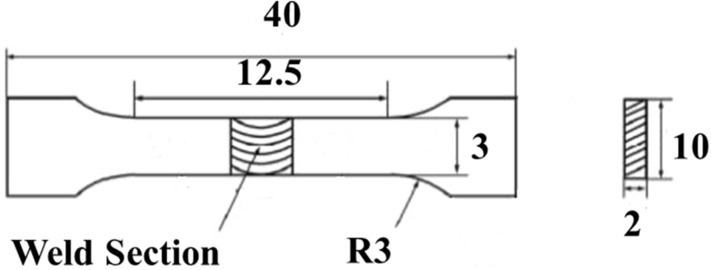
Miniature tensile specimen for AM welds.

Olympus DP25 Optical Microscope (Olympus Corporation, Japan) was used in capturing the microstructures at the base metal (BM), fusion zone (FZ), and the heat affected zone (HAZ). The tensile samples were cut into ASTM E8^22^ subsize for the wrought welds, and a miniature size shown in Fig. 3^23^ was used for the AM welds. Each test sample was pulled using a universal testing machine (UTM) Zwick Roell 2250. TESCAN scanning electron microscopy equipped with energy dispersive spectroscopy (SEM-EDS), available at the University of Johannesburg was used to capture the fractured surface after the tensile test. Indentec Digital Vickers microhardness tester (Indentec, England) at a load of 4.9 N and dwell time of 15 s, ASTM E384^24^, was used in profiling the microhardness across the weld.

## Results and discussion

### Weld bead geometry

**Figure 4:**
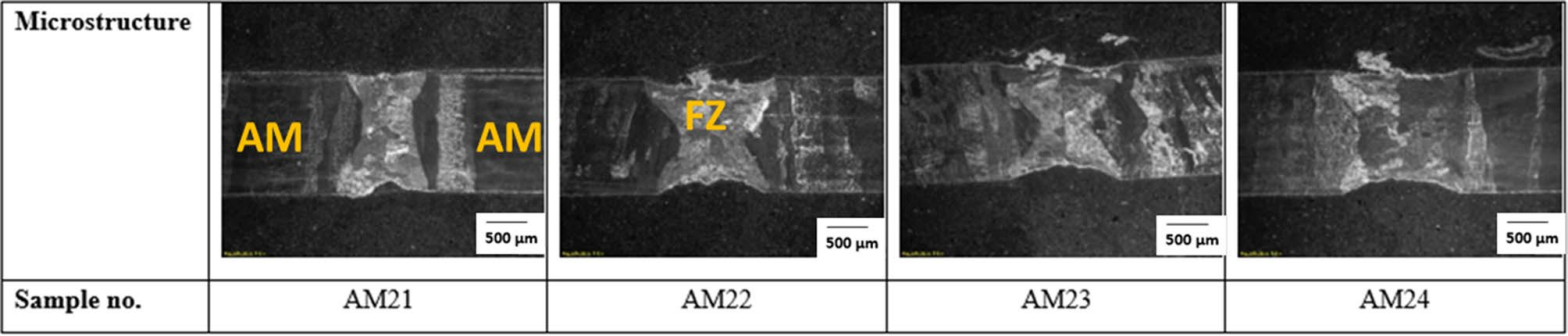
Cross-section of AM welds.

**Figure 5:**
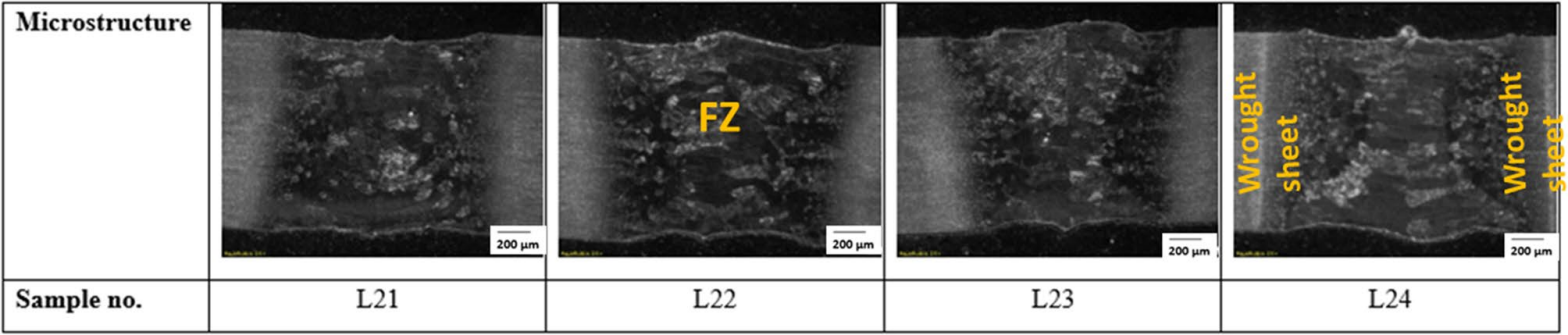
Cross-section of wrought welds.

**Figure 6:**
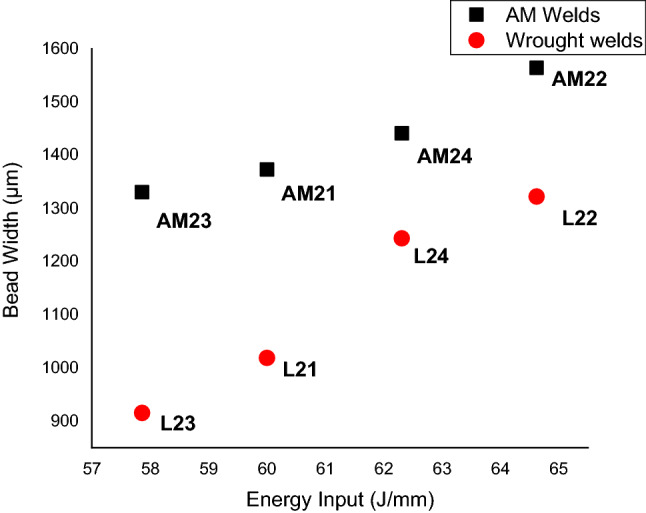
Bead width versus energy input during welding.

The bead geometry was captured using Olympus SZX16 (Olympus Corporation, Japan) macroscope, and stream essentials software was used in measuring the geometry. The weld shape at the fusion zone (FZ) for both the AM and wrought welds appears to have an hourglass shape and full penetration. Furthermore, the cross-section of the AM welds shown in Fig. 4 reveals a wider width of FZ than the wrought welds in Fig. 5, with the average width of AM weld measuring 1426.94 µm and that of wrought welds is 1167.93 µm. The increase in the width of the AM welds could be attributed to the possibility of quicker heat transfer within the AM material because of the higher thermal conductivity of AM material over wrought material. Tavlovich et al.^11^ observed the thermal conductivity of AM material to be 2.5 times that of the wrought material, even up to 800 °C. Energy input also significantly affects the bead width of both the AM and wrought welds, with higher energy input resulting in an increased bead width for all welds, as shown in Fig. 6.

### Microstructure

**Figure 7:**
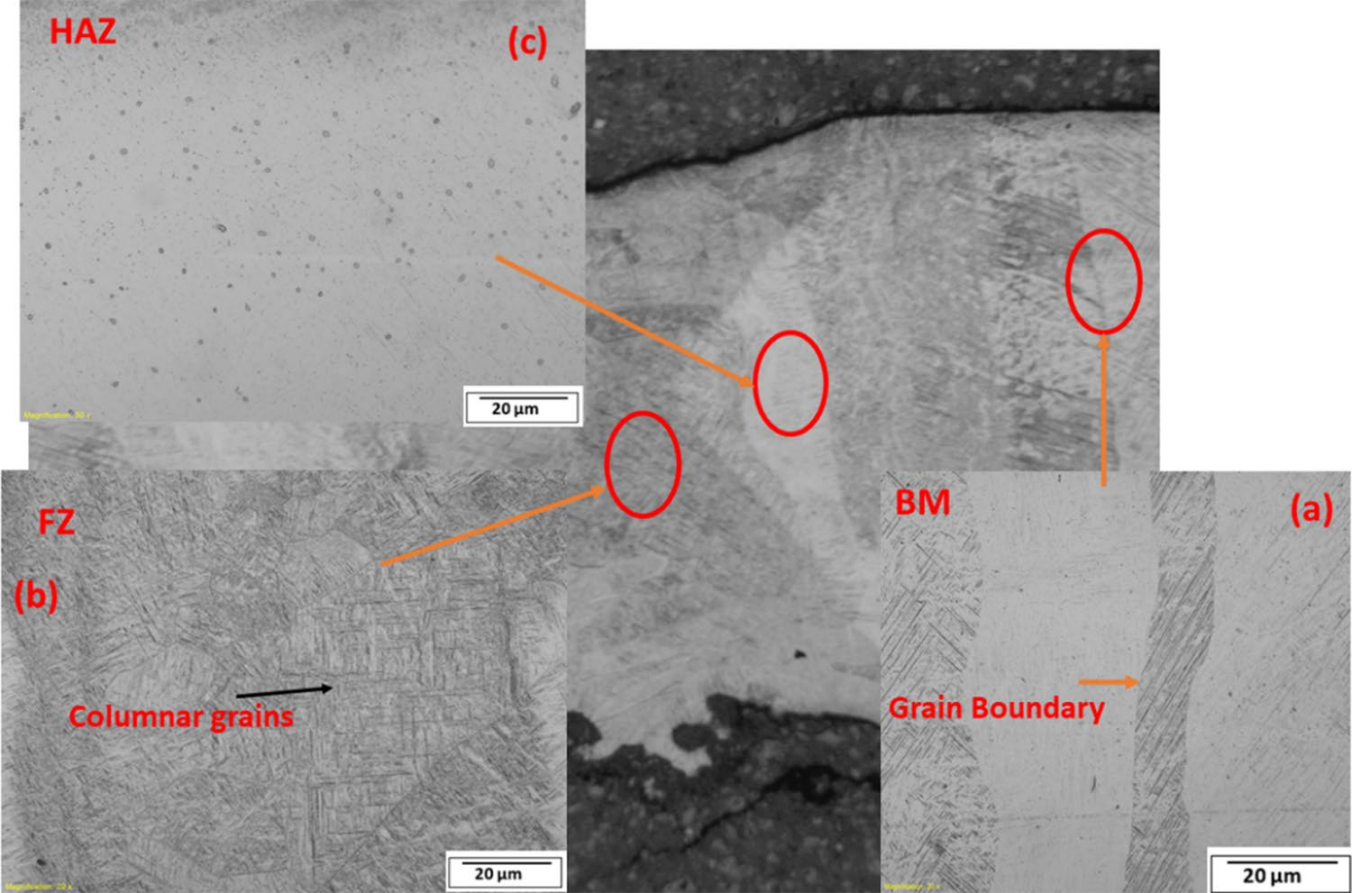
Microstructure of AM welds showing (**a**) base metal, (**b**) fusion zone, (**c**) heat affected zone.

The microstructure of the additive manufactured products is made of the layer-by-layer structure coarse columnar grains of an average length of 85.1 µm, arranged in a sequence of needle-like martensitic α and fine equiaxed grains as also observed by^25–27^ shown in Fig. 7a. The average width of the layer of the columnar grains is ~0.11 mm, and they are as high as the thickness of the material^28,29^. Furthermore, coarse columnar grains with an average length of 49.5 µm exist within the FZ, as shown in Fig. 7b, as a result of cooling from the β phase at a rapid rate above the critical cooling rate of 410 °C/s^30,31^. The shorter length of columnar grains at the fusion zone (FZ), observed after laser welding, is due to increased heat input. Li et al.^32^ explained that increasing heat input on existing columnar grains results in a shorter length of the columnar grains. The heat affected zone (HAZ) in Fig. 7c is made of fine equiaxed grains, which may be due to a reduced temperature below the β transus temperature of 995 °C and a faster cooling rate as a result of lower temperature. Yung et al.^33^ also observed that the lower temperature causes aging of the martensitic microstructure, turning it into fine α particles.

**Figure 8:**
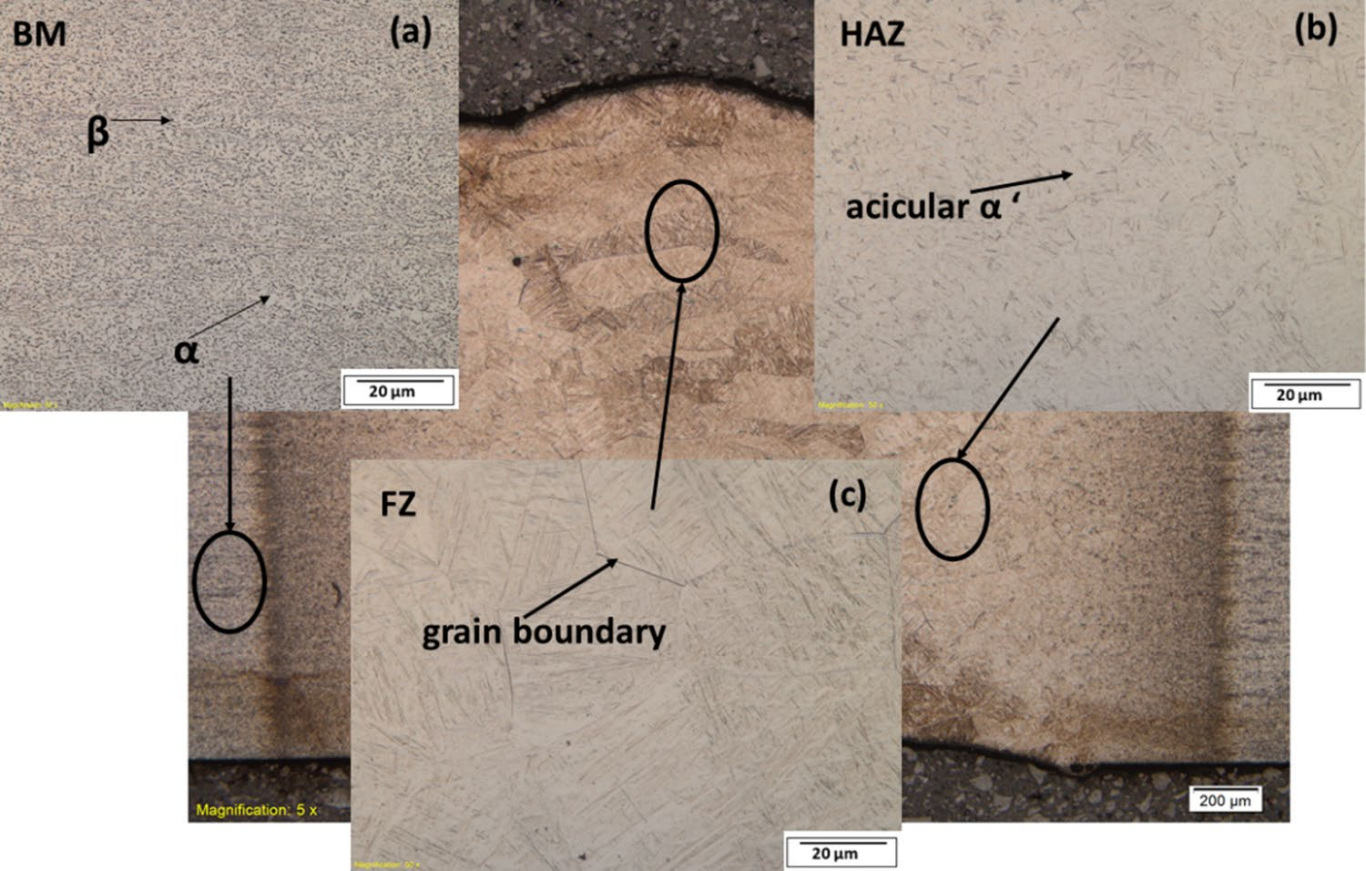
Microstructure of wrought welds showing (**a**) base metal, (**b**) heat affected zone, (**c**) fusion zone.

The BM of the wrought material in Fig. 8a comprises the equiaxed α and β phases with an average grain size of 0.68 µm^6,14,34^. The HAZ in Fig. 8b majorly comprises blocky α and some untransformed β and α phases close to the base metal (BM). The HAZ near the FZ consists of α martensitic microstructure, resulting in higher hardness within the region than the BM. A similar phenomenon was also observed by Kabir et al.^35^. Similar to the FZ of AM welds, coarse columnar grains exist within the FZ of the wrought welds with an average length of 43.3 µm, as shown in Fig. 8c. The grains appear like the needle-like α lamellar and acicular α’, resulting from the zone reaching the liquidus temperature and cooling at a rate above the critical cooing rate of 410 °C/s as explained and also observed in^36–38^. The length of the columnar grains is close to that of the AM welds.

### Microhardness profile

**Figure 9:**
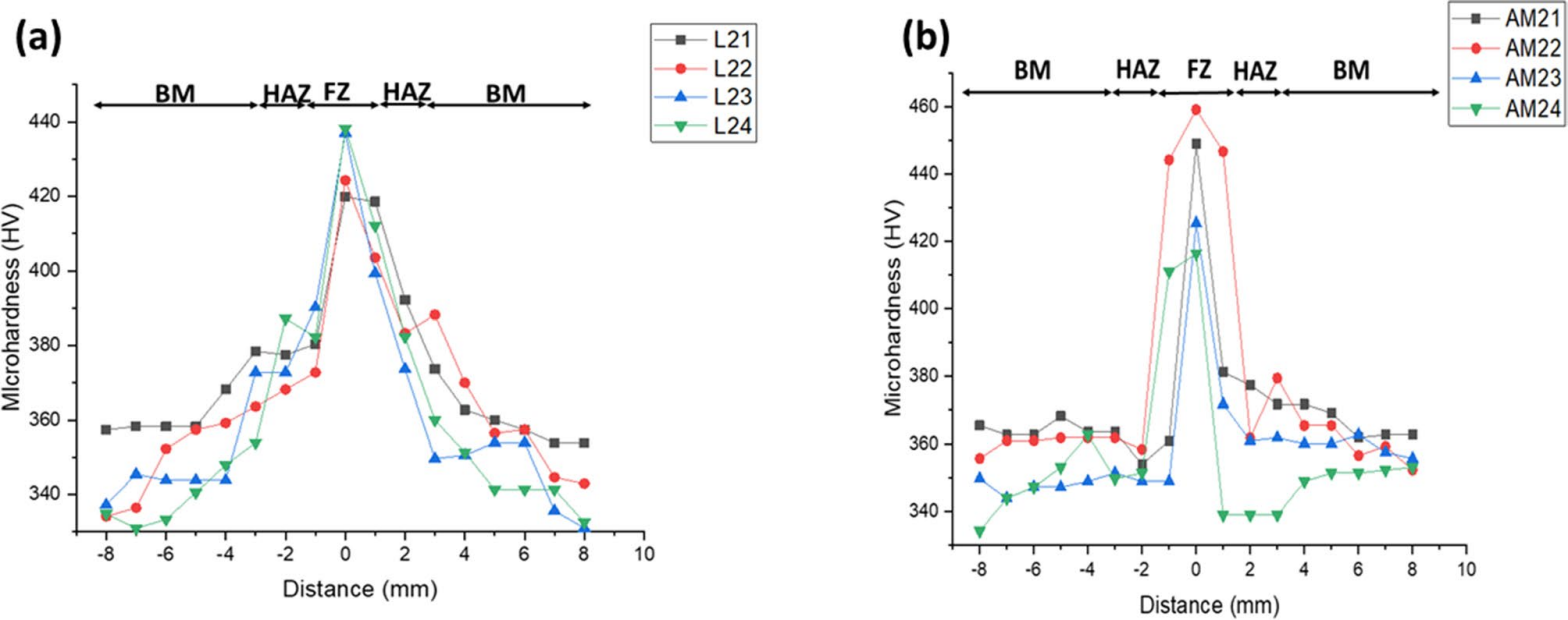
Microhardness profile of (**a**) wrought sheets welds, (**b**) additive manufactured product welds.

The microhardness profile is measured across the weld zone (WZ) and the base metal (BM). Each indentation was done at an interval of 1mm. Results of the wrought welds are shown in Fig. 9a. The FZ has the highest microhardness value ranging from 420 to 438 HV due to the α’ martensitic microstructure, which is similar to observations from^38–41^. Similarly, the heat affected zone (HAZ) is characterized by the coarse α’ martensitic microstructure, with the microhardness ranging from 372 to 392 HV. The BM has an average microhardness value of 350 ± 10 HV, similar to^40–42^ observations.

Figure 9b shows the microhardness profile of the AM welds across the WZ and BM. Similar to the wrought welds, the FZ exhibited the highest microhardness ranging from 416 to 459 HV, followed by the HAZ with microhardness ranging from 339 to 376 HV. The BM shows relatively an almost equal microhardness value with an average value of 357 ± 2 HV. Generally, the microhardness of the FZ and HAZ of the AM welds is higher than that of the wrought welds by an average of 8.5 HV because of the rapid cooling of the already transformed microstructure of the AM products during laser welding. Conversely, the microhardness of the HAZ of the AM welds was reduced by an average of 24.5 HV compared to the wrought welds. This could be attributed to the finer microstructure grains in this zone. Even though the BM's microstructure and that of the FZ are transformed to coarse columnar grains, the FZ's microhardness could be associated with the faster cooling rate experienced during laser welding compared with the cooling rate after the LMD process^42^.

### Tensile strength analysis

**Figure 10:**
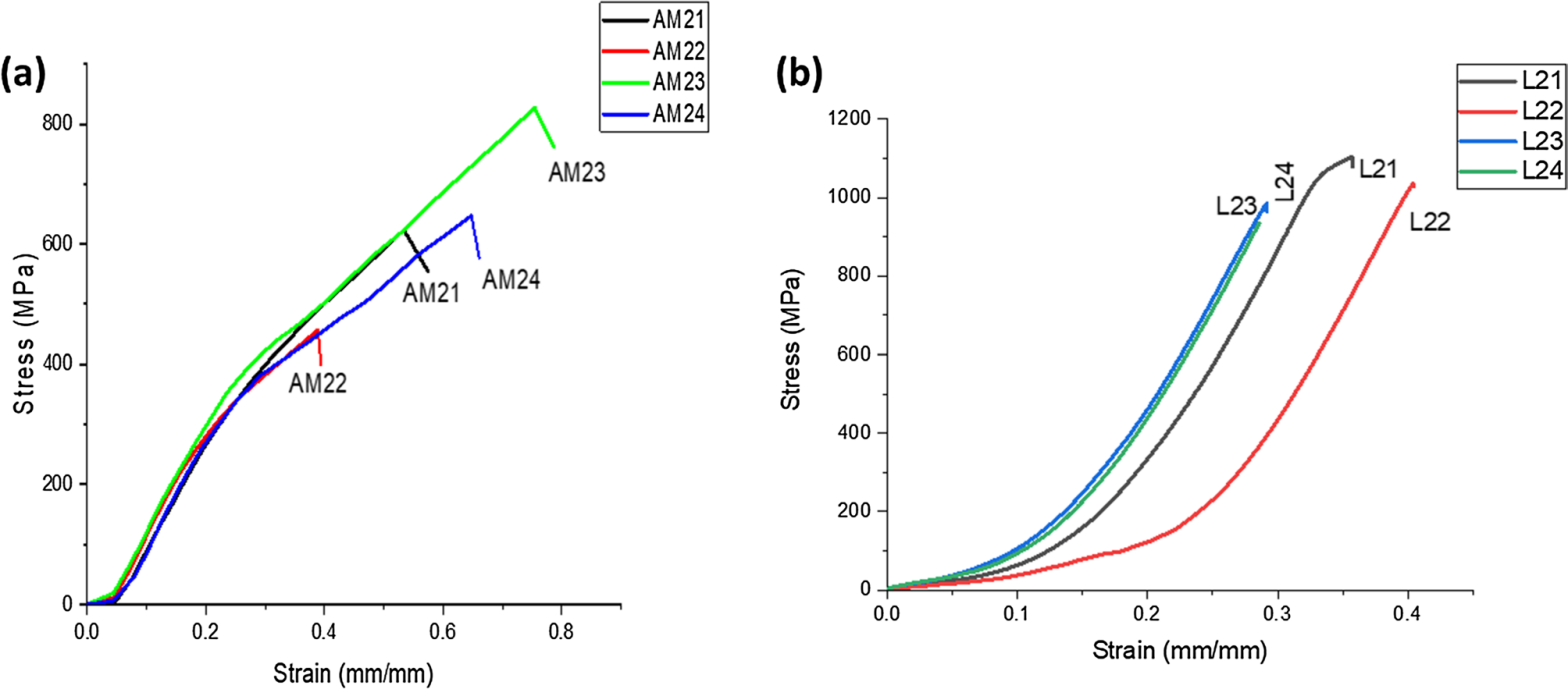
Stress–strain plot (**a**) AM welds, (**b**) wrought welds.

The AM welds show a slight reduction in elongation compared to the parent/base material with a tensile strength of 672 MPa. This is presumably due to the α’martensitic microstructure within the fusion zone (FZ), resulting in reduced ductility in Ti6Al4V as also observed by^43,44^. The maximum stress of the AM welds shown in Fig. 10a was recorded at a stress of 829 MPa for sample AM23 welded using laser power of 2.7 kW and welding speed of 2.8 m/min, with the lowest at 457 MPa, which was welded at a laser power of 2.8 kW and welding speed of 2.6 m/min. In relation to the energy input, there is an improved tensile strength with lower energy input due to a faster cooling rate, which Yung et al.^33^ reported to improve ductility in the weld. Compared with the wrought welds shown in Fig. 10b, there is an average of 50% reduction in ductility in the AM welds, presumably due to the high thermal stress within the FZ. Maximum stress of 1104 MPa was recorded at a welding speed of 2.8 m/min and laser power of 2.8 MPa for the wrought welds. All wrought welds had a tensile strength above the base/parent material with a tensile strength of 950 MPa except sample L24 with laser power of 2.7 kW and welding speed of 2.6 m/min. The wrought welds showed superior strength when compared with the AM welds.

### Fractography analysis

**Figure 11:**
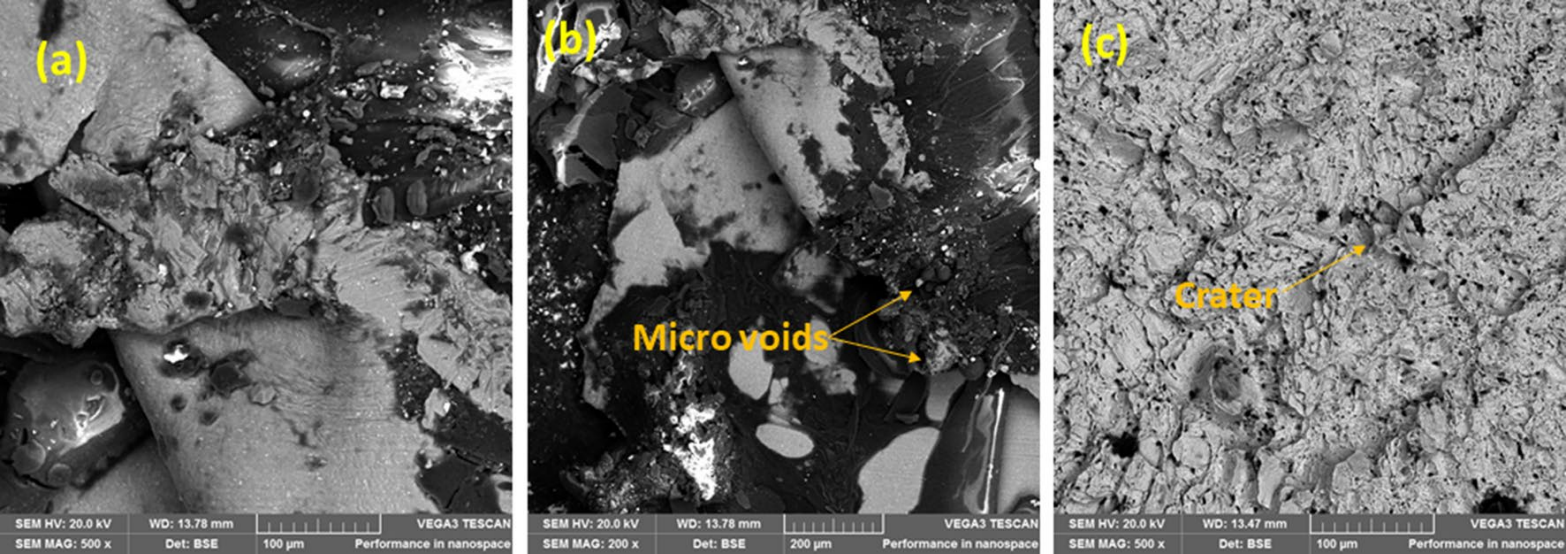
Fractograph of (**a**) LMD Ti6Al4V, (**b**) sample AM22, (**c**) sample L24.

The fractured surface of the additive manufactured product before welding is shown in Fig. 11a. The image show craters that could be presumed to result from a lack of bonding during the manufacturing process. Figure 11b shows the laser welded AM product's fractured surface with a 2.6 m/min welding speed and laser power of 2.8 kW, which exhibited the lowest tensile strength. The presence of microvoids in this sample might have resulted in colossal stress concentration around the voids, thereby leading to failure. Figure 11c shows the fractograph of the wrought welds, which were welded using a laser power of 2.7 kW and a 2.6 m/min welding speed, which exhibited the lowest tensile strength. The presence of crater and inclusions in sample L24 might have failed the material. These craters are one of the primary causes of crack propagation in materials^45–47^.

## Conclusions

The feasibility of joining additive manufactured products manufactured through laser metal deposition (LMD) has been studied. The results evaluated show joint integrity of welded samples to be well above the parent/base material. The following conclusions are drawn:

1. Full penetration was achieved in all welds, and the bead width of the AM welds is wider than the wrought welds due to the higher thermal conductivity of the AM products.
2. The tensile strengths and ductility of the wrought welds are higher than that of the AM welds.
3. The microhardness of the AM welds is higher than the wrought welds.
4. The study shows it is possible to join AM products together with laser welding and still achieve good quality welds.

Future research will address the simulation of temperature distribution during laser welding of AM products manufactured by LMD and the effect of material thickness on the mechanical properties of the weld. Furthermore, the joining of AM products to wrought material will be assessed. Lastly, the effect of stress relieving heat treatment on the manufactured AM products will be considered.

## Data Availability

The data will be made available upon request.
